# Female partners of opioid-injecting men in the Republic of Georgia: an initial characterization

**DOI:** 10.1186/1747-597X-7-46

**Published:** 2012-11-16

**Authors:** Ingunn O Lund, Irma Kirtadze, David Otiashvili, Kevin E O’Grady, Hendrée E Jones

**Affiliations:** 1 Norwegian Centre for Addiction Research, University of Oslo, Oslo, Norway; 2 Addiction Research Center, Alternative Georgia, Tbilisi, Republic of Georgia; 3 Centre for Addictology, Department of Psychiatry, First Faculty of Medicine, Charles University in Prague and General University Hospital in Prague, Prague, Czech Republic; 4 University of Maryland, College Park, College Park, MD 20742, USA; 5 Department of Psychiatry and Behavioral Sciences, Department of Obstetrics and Gynecology, School of Medicine, Johns Hopkins University and RTI International, 3040 E. Cornwallis Road, Research Triangle Park, NC 27709, USA

**Keywords:** Opioid dependence, Intimate female partners, HIV, HCV, Physical violence

## Abstract

**Background:**

HIV and Hepatitis C virus (HCV) infections are strongly related to injection drug use in the Republic of Georgia. Little information is available about HIV and HCV status, sexual risk, support for their partner, and risk for physical violence among the female partners of opioid-injecting men in the Republic of Georgia, many of whom may not be using drugs, yet may be at high risk of being infected with HIV and HCV from their drug-using partners.

**Methods:**

In order to better understand the risks for females whose partners are injecting drugs, the present study conducted an initial investigation of the non-substance-using female partners of 40 opioid-injecting men who were participating in a clinical trial examining the feasibility and efficacy of a 22-week comprehensive intervention that paired behavioral treatment with naltrexone. The 40 female partners were assessed at their male partners’ study intake.

**Results:**

The female sample was 32.3 years old (*SD*=6.7), 37 (93%) were married, with 15.5 years of education. A majority reported at least partial employment the majority of the time during the past 3 years, with only one woman reported being unemployed most of the time during the past 3 years. They self-reported they were 3% HIV-positive and 8% HCV-positive. Their HIV sex risk scores indicated a relatively low risk. However, only 4 (10%) women reported using a condom most of the time while having sex and 15 (38%) report not having had sex during the last 30 days. Experiences of interpersonal violence were common, with 42% reporting physical abuse by their partner during the last year and 48% reporting feeling unsafe in their current relationship.

**Conclusions:**

The alarmingly high rate of failure to use barrier protection methods, together with the high percentage who did not know their HIV and HCV status, suggest that it may be beneficial to include non-substance-using female partners in prevention programs along with their partners to reduce the risk of HIV and HCV spreading from the population of injection-drug–using males into the general population. [This secondary analysis study was funded by an international supplement to the parent randomized clinical trial “Treating the Partners of Drug Using Pregnant Women: Stage II (HOPE)”. ClinicalTrials.gov Identifier: NCT00496990.]

## Background

In the Republic of Georgia there has been a swift and consistent rise in the number of individuals registered as HIV-positive since 1994 [1]. As of September, 2012 there were a cumulative 3,400 HIV-positive cases registered with the National AIDS Centre, with the overall number of people living with HIV/AIDS estimated to be more than 4,000 [2]. Among injection-drug-using adults, the HIV prevalence rate was estimated to be about 1½% in 2010 [3]. This rate is about 2½ times higher than the average prevalence in Europe [4]. The prevalence of hepatitis C (HCV) is estimated to be in the range of 50-70% [5]. This HCV epidemic in Georgia is a major public health concern, and is being driven by injection-drug-using adults. However, other non-drug-using groups have been affected as well, with HCV prevalence in the general population estimated to be 6.7% [6]. Although the overall prevalence of HIV in Georgia is low compared to other former Soviet Union states, the dramatic increase in HIV and AIDS remains a cause for serious concern and there is an urgent need to address the spread of HIV/AIDS. Injection drug use has a central role in the increased HIV prevalence in the region [7]. Prevalence of injection drug use is estimated to be about 1½% in the adult population. Popular injection drugs include heroin and the buprenorphine-mono product, and more recently home-produced amphetamine type stimulants and opioids. The vast majority (98%) of known opioid injectors in Georgia are men. About 60% of all HIV-infected males in Georgia use injection drugs [1].

Many opioid-injecting men are married to illicit-drug-free women, and have never been in drug treatment [8]. While partners and wives of opioid-injecting men in Georgia are exposed to several risk factors resulting from their male partner’s substance addiction, little if any research has been conducted regarding these women. One of the health risks illicit-drug-free women involved with opioid-injecting men face is having their partner infect them with HIV or HCV. Injection drug use is the primary cause of the HIV and HCV epidemics in adult males in the region. Among the majority of men (67.7%) with HIV in the Southern Caucasus region, HIV infection is attributed to injection drug use. In contrast, the majority of women (93.6%) with HIV have been infected through heterosexual contact with an opioid-injecting male partner [7,9,10]. In Georgia more than 70% of the HIV-positive women infected with HIV through heterosexual contact were partners of injection-drug-using males [11].

In any culture, substance use is often accompanied by intimate partner violence [12,13]. In Georgia, this can be deeply influenced by social norms and traditions, and cultural environment that stipulates the asymmetry in gender roles and the marked difference in the level of women’s freedom and independence [14]. In the general population, 9.1 % of women who have ever been married or had an intimate partner have experienced violence from their husbands/partners, and 3.9% of having experienced sexual violence. On the other hand, 78.3% of women think that family problems should only be discussed within a family, and 52.1% of women think that if a man mistreats his wife, others outside the family should not intervene [15]. In addition, it has been suggested that the recent economic development weakened men’s position and, against the normative stereotypes, facilitated women’s rising dominance through increased employment opportunities. “*Having lost their traditional position in the family* (*together with their selfesteem*) *men need to reaffirm their rights and they often do it through drinking and showing their physical power while being drunk*” [15]. Prevalence of male physical aggression towards female partners is approximately six times higher among couples where the man has alcohol or drug problems compared to couples where the man does not have substance abuse problems [16]. Opioids may not typically lead to more aggression [12], but multi-substance use is common among individuals that use opioids [17] and the majority of injecting-drug-using adults in Georgia seem to use poly-substances [5,18]. This research would strongly suggest that Georgian women in relationships with opioid-injecting men are likely to be at increased risk of experiencing intimate partner violence.

The present secondary analysis study has two primary aims. The first aim was to examine HIV and HCV prevalence among the illicit-drug-free female partners of opioid-injecting men in the Republic of Georgia. The second aim was to examine the prevalence of interpersonal violence and feeling unsafe in their current relationship among these women.

## Methods

### Setting

The study was conducted at the Alternative Georgia ( http://www.altgeorgia.ge/?lang=2) field site. This independent nonprofit research institution is located in a central district of Tbilisi, Georgia.

### Participants

To describe the participants in the present study, we first describe their opioid-injecting- male partners, who were the primary focus of the parent study [2]. Male participants were recruited by flyers, word-of mouth, and advertisements given to harm reduction programs and hospital staff. Face-to-face screening interviews on the research site were conducted. A total of 74 men between May 2006 and January 2009 were screened. Of these men, 19 failed to show for their initial intake appointment, while 55 were evaluated for eligibility on the following criteria: 18 years or older, having a current illicit-drug-free intimate female partner with whom they had regular contact, lack of current suicidal ideation, meeting DSM-IV criteria for current opioid dependence, not meeting DSM-IV criteria for thought disorder and free of cognitive impairment that prevent them from completing the study [19,20]). Men who indicated current physical abuse of their female partner to an extent that might be life threatening were excluded from the study. Men who indicated no current physical abuse towards their female partner, or physical abuse that would not be deemed to cause serious physical harm, were included in the study. Following successful administration of baseline screening instruments, 20 men were randomized to behavioral treatment+naltrexone and 20 men to “usual care”. All 40 men were then asked to provide a blood sample to test for HIV and HCV. One of the men (3%) was HIV-positive, 22 (55%) were HIV negative, 13 (33%) refused to provide blood for testing and 4 (10%) dropped out of the study; 24 (60%) men tested positive for HCV, 5 (13%) men tested negative, 7 (18%) refused to provide blood for testing, and 4 (10%) then dropped out of the study [19].

After their opioid-injecting male partners had consented to participate in the study and had been screened the female partners were informed about the study by their male partners. The female partners were invited to visit the research site and sign a written informed consent. All 40 female partners visited and consented to participate in the study, and all self-reported no prior history of illicit drug use as part of a baseline intake that also collected demographic data. All female participants received cash equivalent of $18 US for their interview.

The behavioral treatment plus naltrexone intervention for the male participants utilized counseling sessions with Motivational Interviewing for both the male participant and the couple, monetary rewards for drug abstinence, and detoxification followed by naltrexone treatment. Male participants in the usual care treatment arm were provided once-a-week individualized drug education sessions together with referrals to detoxification and aftercare, at their request.

The project was approved by the Institutional Review Boards of the Georgian HIV/AIDS Patients Support Foundation and Johns Hopkins University.

### Measures

All measures were translated into Georgian, and then independently back-translated into English to determine accuracy of the initial translation. Questions were examined for cultural appropriateness and were adjusted to reflect the Georgian local socio-cultural context, such as specifics of local education system, income rates, illicit drugs availability, and so forth. Any final adjustments to each measure were undertaken following examination of the original scale and the back-translation.

### The Baltimore risk assessment battery (BRAB)

To measure HIV drug-risk, a modified version of the HIV Risk Assessment Battery (RAB) was used, the Baltimore Risk Assessment Battery [21,22]. The BRAB sex-risk score included 9 questions: (1) sexual orientation [score: 0 (Heterosexual) or 1 (bisexual)], (2) number of male partners, (3) number of female partners, (4) frequency of exchanging sex for drugs, (5) frequency of exchanging drugs for sex, (6) frequency of exchanging sex for money, (7) consistency of condom use, (8) frequency of exchanging money for sex, and (9) frequency of sex with someone of known HIV/AIDS positive status. Questions (2)-(9) used 4-point rating scales, scored 0–3. Possible scores range from 0 to 25, inclusive, with higher scores indicating greater HIV sex-risk behavior.

### Partner support questionnaire (PSQ)

The PSQ, based on the self-report Norbeck Social Support Questionnaire [23], is a 16-item questionnaire, with each item scored on a 5-point scale, from 0 = “never” to 4 = “very often”. It asks the respondent to rate herself on various aspects of her support for her partner in reaching and maintaining drug abstinence (e.g., “Do you compliment your partner on not using heroin”; “Do you help your partner to think of things to do other than using heroin”). Possible scores ranges from 0 to 64, inclusive, with higher scores indicating greater support.

Two additional binary items were added to the end of the PSQ: “Have you been hit, kicked, punched, or otherwise hurt by your partner within the past year? (past month for follow-up assessments)” and “Do you feel safe in your current relationship?’ which were not scored as part of the PSQ. These two items were examined separately in order to assess if the women had experienced interpersonal violence during the year prior to the interview and whether they felt safe in their current relationships.

### Statistical analyses

Descriptive information on background characteristics, HIV and HCV status, sex risk, physical abuse, and feeling unsafe in the current relationship is presented in terms of frequencies and percentages or means and standard deviations.

## Results

### Participant characteristics

On average, the women, all of whom were Caucasian, the majority of whom being native Georgian (88%), with the remaining 12% nationals of neighboring countries (Russia, Armenia, and Azerbaijan), were 32.3 years old, 37 (93%) were married, with an average 15.5 years of education (Table 1). A majority reported at least partial employment the majority of the time during the past 3 years. Only one woman reported being unemployed most of the time during the past 3 years. The most common employment occupation was business manager (33%) followed by clerical/sales and semiskilled, each reported by 13%.

**Table 1:** **Background Characteristics for illicit-drug-free female partners of opioid-injecting men in the Republic of Georgia (*N*=*40*)**

	***M *(*SD*)**	***f *(%)**
Age	32.3 (6.7)	
Education (years)	15.5 (2.2)	
Married		37 (93%)
Usual employment last 3 years:		
Full-time student (unemployed)		3 (8%)
Housewife		12 (30%)
Unemployed (seeking job)		1 (3%)
Part time (<35 hours per week)		13 (33%)
Full time		10 (25%)
Irregular hours		1 (3%)
Usual or last occupation:		
Higher executives		1 (3%)
Business managers		13 (33%)
Administrative Personnel		4 (10%)
Clerical and Sales		5 (13%)
Skilled Manual		2 (5%)
Semiskilled		5 (13%)
Unskilled		1 (3%)
Never worked		9 (23%)
Currently employed		19 (48%)

### Prevalence of HIV and HCV

HIV and HCV status of the women participants was determined by self-report. Positive HIV status was reported by one woman. She indicated that she was infected with HIV following sexual contact with her husband who got infected in prison due to sharing syringes. Negative HIV status was reported by 20 women and 15 reported they were unaware of their HIV status (Table 2). Positive HCV status was reported by 3 women, 24 reported negative HCV status, and 9 women reported they were unaware of their HCV status. Four female participants dropped out of the prior to the self-reported assessment of their HIV and HCV status. These 4 women participants were the partners of the men who had refused to provide a blood sample for testing and dropped out of the study.

**Table 2:** **HIV and Hepatitis C status among illicit-drug-free female partners of opioid-injecting men in the Republic of Georgia (*N*=40)**

	***f *(%)**
HIV status	
Positive	1 (3)
Negative	20 (50)
Unknown	15 (38)
Drop-out	4 (10)
Hepatitis C status	
Positive	3 (8)
Negative	24 (60)
Unknown	9 (23)
Drop-out	4 (10)

Given the relatively large percentage of the sample unaware of their HIV and HCV status, answers to these two questions were examined for their male partners. Of the 15 women who did not know their HIV status, 13 of their male partners had refused to provide blood for testing, while of the 9 women who were unaware of their HCV status, 7 of their male partners refused to undergo HCV testing.

### Sex risk

Because of the lack of variability in responding to the items on this scale (see following), no internal consistency reliability for the BRAB HIV sex risk scale is reported.

The BRAB HIV sex risk score (Table 3) does indicate a relatively low risk for the women. All women reported themselves to be exclusively heterosexual, and no women reported she had exchanged sex for drugs or money, or exchanged drugs or money for sex. However, only 4 (10%) women reported using a condom most of the time while having sex and 15 (38%) report not having had sex during the last 30 days. Of these 15 women, 7 (47%) of their male partners indicated that they had not had sex in the last 30 days, with 2 of the remaining 8 men indicating they used condoms “all of the time”, 1 “most of the time”, and 5 “some of the time”. One woman reported having HIV-positive male partner (one of the male participants in the parent study was HIV-positive), and indicated that she had used condoms “most of the time” in the last 30 days. Twenty women (51%) were not at all worried and 7 (18%) were considerably or extremely concerned about getting HIV/AIDS.

**Table 3:** **Sex-risk, partner support, and physical abuse and safety among illicit-drug-free female partners of opioid-injecting men in the Republic of Georgia (*N*=40)**

	***M *(*SD*)**	***f *(%)**
Baltimore Risk Assessment Battery (BRAB) ^†^		
Total sex risk scale score	3.6 (1.7)	
*How often used a condom while having sex the past 30 days*		
Have not had sex the last 30 days		15 (38)
Some of the time		21 (53)
Most of the time		4 (10)
*How worried about getting HIV*/*AIDS*^‡^		
Not at all		20 (51)
Slightly/moderately		12 (31)
Considerably/extremely		7 (18)
Partner support questionnaire (PSQ) *	41.0 (5.9)	
Hit/kicked/punched by partner in the last year		17 (42)

^†^Sex Risk scale has a possible range of 0–25, inclusive.

^‡^One participant was missing data for this question.

*PSQ had a possible range of 0–64, inclusive.

### Partner support

The PSQ had an internal consistency reliability α = .78 in this sample, indicating that the scale score could be considered a reliable measure of partner support.

The women generally believed they often provided support (see Table 3), with scores ranging from 22 to 50, inclusive. Assuming the theoretical midpoint of the scale would be 32 (an average item score of 2 = “sometimes”), only 3 women provided support that might be seen as less than adequate.

### Physical abuse and safety

Physical abuse by their partner within the last year was reported by 42% of the women, and 48% said they felt unsafe in their current relationship.

## Discussion

Female partners of injection-drug-using men in the Republic of Georgia are at high risk for contracting HIV and HCV [9]. This is the first study that describes the prevalence of HIV and HCV among this recently identified high-risk group of illicit-drug-free female partners of opioid-injecting men in the Republic of Georgia, almost all of whom were married.

When asked about their HIV status only one woman reported that she was HIV-positive, half said they were HIV negative, and more than one third stated they were never tested. A small number of women reported being infected with HCV, almost two-thirds said they were HCV-negative, and close to one in four stated they were unaware of their status. It has been reported that stigma against and fear of discrimination has led many people in Georgia to conceal a positive HIV status [24]. This situation might be the case for some of the women in the present study as well, and may extend to revealing HCV status. Kirtadze et al. [25] examined the attitudes and beliefs of 34 health service providers in Georgia who had contact with injection-drug-using women. These providers indicated less tolerance towards such women, believing they were failures as a good mother, wife, or child. The authors concluded that drug-using women in Georgian culture were “twice stigmatized” – as failed mothers/wives/daughters, and as less acceptable than men, even drug-using men.

In addition, the parent study records suggest that male participants refused HIV testing due to the fear of being diagnosed positive (e.g., “*It is better if I do not know if I am infected*”), and refused HCV testing due to lack of available treatment options (e.g., “*even if I am positive there is no treatment that I can afford*”). This might well explain low motivation of participants, both, males and females, to know their health status.

Half the women reported not being worried about contracting HIV/AIDS and less than one in five reported being considerably or extremely worried about being infected with HIV/AIDS. The low prevalence of concern is surprising since a majority of women with HIV in Georgia have been infected from a partner who injects drugs [9]. The limited understanding of and attitude towards HIV/AIDS in Georgia suggests that there may be a serious lack of available information about risk factors for HIV infection [6]. Alternately, there may be a taboo among Georgian women to express worry about contracting HIV/HCV, due to the heavy social stigma associated with these infections. Moreover, the fear of being arrested and a lack of trust in health care providers contribute to the fact that only about 5% of injection-drug-using adults in Georgia are tested for HIV on a regular basis [7]. Therefore, it may be the case that the prevalence of HIV is notably higher among injection-drug-using adults than what has been estimated.

Moreover, despite a low average sex risk score, only 1 in 10 women reported that her partner used a condom most of the time. The women may be influenced by the widespread belief among Georgian women that if they are not injecting drugs or are sex workers, they are not at risk of HIV or HCV infection [7]. Safe sex would be a protective factor against HIV and HCV for the women. However, other studies have also found that sexually active women in Georgia rarely use or discuss using condoms with their partners [24]. More than a third of the women reported not having had sex during the last 30 days. These results merit further research to determine the extent that low rates of sexual activity are related to the slow spread of HIV and HCV among women in the Republic of Georgia. Previous research has revealed a low level of knowledge of HIV risk factors and high prevalence of unsafe sex among the general population in Georgia [6]. Inadequate knowledge of HIV risk factors and methods for HIV risk reduction seem to be a widespread problem in Georgia [6]. Results of the present study would suggest that unsafe sex frequently occurs in an extremely high-risk population, non-drug-using female partners of injection-drug-using men.

High-risk injection practices are common in the Republic of Georgia. Previous research reported that in some subgroups more than four out of five opioid-injecting drug-using men in Georgia are sharing needles with others [26,27]. Thus, even if the opioid-injecting male partners of illicit-drug-free females are not having sex with other women or men, the male partner remains at high risk of getting infected with HIV and HCV and passing these infections to his female partner through sexual contact.

The women generally perceived themselves as supportive of their partners’ efforts to reach and maintain abstinence.

Seventeen women (43%) had experienced physical abuse by their partner within the last year prior to the interview and close to half reported feeling unsafe in their current relationship. In a study including more than 1,000 women in the general population in Georgia, 6% reported experiencing physical violence and 11% sexual violence during the last five years. This study measured all physical and sexual violence, not just violence by partner [28]. Another study including close to 8,000 women in Georgia reported that 2% had experienced physical abuse and 3% sexual abuse by a partner during the last 12 months [27]. Females in a relationship with an opioid-injection drug-using male partner seem to be at increased risk of experiencing interpersonal violence compared to the general population of women in Georgia. Another health risk is that the drug-injecting man will initiate his partner into drug use. Women commonly start using drugs because of their partner [29,30]. Preventing female partners of opioid-injecting men from initiating drug use is a critically important goal. Including these women in their partner’s treatment program might enable them to improve their relationship with their partner, learn how to resist the use of illicit drugs, and better support their partner in his rehabilitation.

### Study limitations

First, the sample size of the present study was relatively small. Therefore, the findings need to be interpreted with restraint. Second, selection bias that attracted motivated males in a stable relationship with an interest in treatment might have operated to yield a sample of men who are more likely to engage in safe sex practices. It is difficult to determine the extent to which this statement might be true, given the high non-response rate regarding HIV and HCV infection for the male as well as the female participants. Selection bias may have been further compounded by attrition. Thus, the small sample size and the impact of both selection bias and attrition may have adversely impacted the ability to determine precise estimates of HIV and HCV in the population of non-substance-using female partners of injection-drug-using men. Third, a relatively large number of men who expressed an interest in participation in the parent study failed to enter the study: 19/74 (26%) failed to show up for their intake appointment, and 15/74 (20%) were determined to be ineligible. Because information on the women in the present study is gathered from the female partners of the male participants, such a relatively high rate of participant loss potentially limits the generalizability of the findings. However, the rate of non-participation is not unexpected, because the parent study focused on attracting non-treatment-seeking men into treatment. Fourth, stigma and the attendant fear of disclosure may have operated to restrict the range of responding and increase the chances of socially desirable responding, also limiting our ability to generalize our findings. Fifth, HIV and HCV status was based on self-report measures. Thus, the reliability of such data depends on truthful answers from respondents. Sixth, there was a relatively large amount of missing data on for the questions regarding HIV and HCV status, because the women failed to answer these questions. Being either HIV-positive or HCV-positive is associated with considerable stigma in Georgia, and fear of stigmatization may have influenced some respondents to refuse to answer these questions – or, potentially to answer in socially desirable ways. Finally, information on sexual violence or abuse independent of physical violence was not collected.

### Strengths of the study

Research on non-opioid-injecting female partners of opioid-injecting men is scarce, both in the Republic of Georgia and elsewhere [31]. Information about HIV status, HCV status, sex risk behavior, concern about getting HIV and interpersonal violence may prove invaluable for designing HIV interventions for injection-drug-using men and their female partners. We can envision that public health efforts should be focused in two directions. First, efforts need to be made to provide additional information to potentially sexually active Georgian women – particularly female partners of injection-drug-using men – that barrier protection methods are the best protection against HIV and and HCV infection. Second, providing couples therapy in some form, even if only for 2–3 sessions, may allow for direct outreach and education of both members of the couple on the risks of HIV and HCV infection.

## Conclusions

In settings where prevalence of HIV/AIDS is low, the epidemic is mostly concentrated within specific groups, as is the case of Georgia, in injecting-drug-using adults. Preventive interventions should target populations at risk for HIV transmission. Such targeted evidence-based interventions have maximal impact in terms of both preventing the spread of infection and allowing for the most efficient allocation of limited financial resources. A high proportion of the women in the present study had never been tested for HIV and HCV, and reported no motivation to do so when offered. This, along with the low reporting of condom use, strongly indicate the need for targeted education efforts that would provide information about disease development and treatment options, and would encourage this population to seek appropriate counseling and testing. Prevention programs should educate both the injection-drug-using male and his female partner in the risks of HIV and HCV transmission from unprotected sex, and thereby to reduce the risk of HIV and HCV transmission from the population of injection-drug–using individuals into the general population. These women are also at alarming risk compared to the general population of women for experiencing interpersonal violence.

## Competing interests

The authors declare they have no competing interests.

## Authors’ contributions

HEJ designed the initial study and protocol, while DO and IK provided input into the refinement of the protocol. DO was Principal Investigator at the Republic of Georgia site where the protocol was implemented and was responsible for the medical screening and naltrexone treatment and monitoring. IK was responsible for data collection and patient management. KEO’G conducted the data analyses. IOL wrote the first draft of the manuscript. All authors discussed the results and implications and commented on the manuscript at all stages. All authors have approved the final manuscript. No honorarium, grant, or other form of payment was given to any author or any other individual to produce the manuscript.
